# Metabolic health is more closely associated with decrease in lung function than obesity

**DOI:** 10.1371/journal.pone.0209575

**Published:** 2019-01-23

**Authors:** Hea Yon Lee, Hae Kyung Yang, Ho Jin Song, Hee Jae Chang, Ji Young Kang, Sang Haak Lee, Solji Han, Young Kyoon Kim

**Affiliations:** 1 Department of Health Promotion Medicine, College of Medicine, The Catholic University of Korea, Seoul, Korea; 2 Department of Internal Medicine, College of Medicine, The Catholic University of Korea, Seoul, Korea; 3 Division of Pulmonary Medicine, Department of Internal Medicine, College of Medicine, The Catholic University of Korea, Seoul, Korea; 4 Department of Applied Statistics, Yonsei University, Seoul, Korea; Weill Cornell Medical College in Qatar, QATAR

## Abstract

**Objective:**

Previous studies have evaluated the link between metabolic syndrome and obesity with impaired lung function, however findings have been controversial. We aimed to compare lung function among subjects with different metabolic health and obesity status.

**Methods:**

Total 10,071 participants were evaluated at the Health Promotion Center in Seoul St. Mary’s Hospital between January 2012 and December 2014. Being metabolically healthy was defined as having fewer than three of the following risk factors: high blood pressure, high fasting blood glucose, high triglyceride, low high-density lipoprotein cholesterol and abdominal obesity. Obesity status was defined as body mass index (BMI) higher than 25 kg/m^2^. Analyses of pulmonary function were performed in four groups divided according to metabolic health and obesity: metabolically healthy non-obese (MHNO), metabolically health obese (MHO), metabolically unhealthy non-obese (MUHNO), and metabolically unhealthy obese (MUHO).

**Results:**

Metabolically unhealthy subjects were more prone to decreased lung function compared with their metabolically healthy counterparts, regardless of obesity status. When multinomial logistic regression analysis was performed according to quartiles of forced vital capacity (FVC) or forced expiratory volume in 1 second (FEV_1_) (% pred), after adjusting for age, sex, and smoking status, odds ratio (OR) for the lowest FVC and FEV_1_ (% pred) quartiles were significantly higher in MUHO subjects (1.788 [95% CI, 1.531–2.089] and 1.603 [95% CI, 1.367–1.881]) and lower in MHO subjects (0.768 [95% CI, 0.654–0.902] and 0.826 [95% CI, 0.700–0.976]) with MHNO group as the reference, when OR for highest FVC and FEV_1_ quartiles were considered as 1.0

**Conclusion:**

Metabolic health is more closely associated with impaired lung function than obesity.

## Introduction

Obesity is the main cause of various metabolic diseases leading to an increase in risk of cardiovascular disease such as coronary, cerebrovascular, peripheral arterial disease and heart failure [1,2]. It has been reported that approximately 60% of obese individuals have metabolic syndrome (MetS) in the US [3]. The prevalence of MetS was approximately 35% in the adult population of US during 2003–2012, meanwhile the prevalence of MetS in Korea is steadily increasing up to 28.9% in 2013 [4].

Nevertheless, recently subgroups of obesity that have a good metabolic profile have gained much interest. This group is referred to as the “metabolically healthy obesity (MHO)”, which indicates obese objects without satisfying the criteria for being metabolically unhealthy such as elevated blood pressure, dyslipidemia, abdominal obesity, insulin resistance, or elevated surrogate markers of systemic inflammation [5,6]. Previous studies show that metabolically unhealthy obese phenotypes tend to have higher risk of all-cause or cardiovascular mortality compared with their metabolically healthy counterpart [7–10]. However, the clinical significance of MHO group has not been widely examined.

Obesity is known to interfere with respiratory function by decreasing lung volume and lung compliance [11,12]. MetS is also identified as an independent risk factor for greater lung function impairment and worsening respiratory symptoms [13–15]. Therefore, both obesity and metabolic health, and their interactions, should be considered to estimate the risk of lung function impairment. The combination of obesity and MetS seems to impair lung function even further, yet it is unclear how much of the MetS related lung effects occur independently of obesity and vice versa [15].

Therefore, we aimed to compare lung function between four groups, divided by obesity status using BMI and metabolic health in a large, health-screening cohort.

## Material and methods

### Study subjects

We collected and retrospectively reviewed the data from 10,071 subjects who participated in a medical health check-up at the Health Promotion Centre in Seoul St. Mary’s Hospital, a 1200-bed tertiary university teaching hospital between January 2012 and December 2014. The study was approved by the institutional review board of Seoul St. Mary’s Hospital, which permitted evaluation and publishing of information from the individual’s records. The requirement for informed consent was waived because of the retrospective nature of the study.

The study population were all Korean by ethnicity and age range was from 19–85 years. The purpose of the medical health checkup was to promote regular health checkups and to facilitate early detection of existing diseases. The parameters measured were basic hematologic tests (complete blood count, blood chemistry), urinary tests, lung function test, chest x-ray, abdominal sonogram and gastroscopy. The examinations were performed by MDs (gastroenterologist and radiologists), trained nurses and workers.

Inclusion criteria were age ≥ 19 years, data to assess lung function and presence of MetS. Medical information was gathered through a standardized questionnaire (S1 File) and we excluded subjects with chronic obstructive lung disease, asthma, infectious lung disease such as bacterial pneumonia and tuberculosis, interstitial lung disease, occupational lung disease, lung cancer and severe cardiovascular disease. Repeated visits of the same person were omitted and only the data from the first visit was analyzed.

### Lung function

Spirometry was performed as recommended by the ATS/ERS guidelines [16] using Vmax 2130 (SensorMedics, Yorba Linda, CA, USA). Absolute values of FVC and FEV_1_ were obtained, and the percentage predicted values (% pred) for FEV_1_ and FVC were calculated from the following equations obtained in a representative Korean sample [17].

$$\text{Predicted}\;\text{FVC}=-4.8434–\left(0.00008633{\times \text{age}}^{2}\left[\text{years}\right]\right)+\left(0.05292\times \text{height}\;\left[\text{cm}\right]\right)+\left(0.01095\times \text{weight}\;\left[\text{kg}\right]\right)$$

$${\text{Predicted}\;\text{FEV}}_{1}=-3.4132–\left(0.0002484{\times \text{age}}^{2}\left[\text{years}\right]\right)+\left(0.04578\times \text{height}\;\left[\text{cm}\right]\right)$$

We also analyzed the patients according to the quartiles of FVC or FEV_1_ (% pred).

### Anthropometric measurements and blood tests

Height, weight and waist circumference were measured through a bioelectrical impedance method using Inbody 720 (Biospace, Seoul, Korea). Body mass index (BMI) was calculated by weight (kg) divided by the squared value of height (m) (kg/m^2^). Blood pressure was measured using an automatic BP monitor (TM-2655P; P.M.S, Berkshire, UK), after five minutes of rest. Using Inbody 720, body composition values including body fat mass (kg) and percentage (%), skeletal muscle mass (kg), and waist-hip ratio (WHR) were measured.

Blood samples were collected in all of the subjects after an overnight fast and centrifuged within 30 minutes. Samples were collected in sodium fluoride tubes for plasma glucose measurement and in serum-separating tubes for others. All of the samples were analyzed at the central laboratory in Seoul St. Mary’s hospital. Serum fasting glucose levels were measured via the hexokinase method; fasting triglyceride, total and low-density lipoprotein (LDL) cholesterol levels were measured via enzymatic assays; and high-density lipoprotein (HDL) cholesterol levels were measured via selective inhibition. All measurement were taken using a Hitachi 7600 autoanalyzer (Hitachi Ltd., Tokyo, Japan). HbA1c was measured by high-performance liquid chromatography using DCCT-aligned methods (Tosoh-G8, Tosoh, Tokyo, Japan).

The presence of diabetes mellitus was determined by answers to the participant self-questionnaire and the diagnostic criteria of the American Diabetes Association [18]. The presence of hypertension was defined as blood pressure (BP) ≥140/90 mm Hg or presently taking anti-hypertensive medication, according to criteria [19]. Smoking status was determined by the questionnaire. A smoker was defined as a subject who had ever at least five packs of cigarettes in his life. Subjects were categorized as non-smokers, ex-smokers, or current smokers.

### Definition of being metabolically healthy obese

Obesity phenotypes were defined based on BMI category based on the revised Asia-Pacific criteria of obesity in Asian populations (non-obese <25 kg/m^2^, obese ≥25 kg/m^2^) [20].

Being metabolically healthy was defined as having less than three of the following risk factors, using the diagnostic criteria for MetS [21]:

1. Systolic blood pressure ≥ 130 mmHg and/or diastolic blood pressure ≥ 85 mmHg, or on antihypertensive treatment.
2. Fasting glucose ≥ 100 mg/dl or being treated for diabetes.
3. Waist circumference; men ≥90 cm, women ≥ 85 cm for Koreans [22]
4. Triglyceride ≥ 150 mg/dl.
5. HDL-cholesterol <40 mg/dl in men, <50 mg/dl in women.

According to these criteria, participants were divided into 4 groups:

1. Metabolically healthy, non-obese (MHNO): BMI <25 kg/m^2^ and <3 metabolic risk factors.
2. Metabolically healthy, obese (MHO): BMI ≥25 kg/m^2^ and <3 metabolic risk factors.
3. Metabolically unhealthy, non-obese (MUHNO): BMI <25 kg/m^2^ and ≥3 metabolic risk factors.
4. Metabolically unhealthy, obese (MUHO): BMI ≥25 kg/m^2^ and ≥3 metabolic risk factors.

### Statistical analysis

Continuous variables were presented as means ± standard deviation and analyzed among the four groups by one-way analysis of variance (ANOVA) test and post hoc analyses with the Tukey’s b method, and analysis of covariance (ANCOVA) test was performed to adjust for age and sex. Categorical variables were presented as frequencies and percentages, and analyzed using Pearson’s chi-squared test for discrete variables. Multinomial logistic regression analyses with the quartiles of FEV_1_ or FVC (% pred) as the dependent variable were performed after adjusting for confounding factors such as age, sex, smoking, skeletal muscle mass and body fat mass included in the model. All tests were two sided and *p* values < 0.05 were considered to be statistically significant. All analyses were performed with the SPSS computer package (version 18.0; SPSS Inc., Chicago, IL, USA).

## Results

### Clinical characteristics of the study population

The clinical characteristics of the participants are shown in Table 1. Among 10,071 participants, the mean age was 48.7 years (range 19–93 years) and 64.3% were male. Mean value of BMI was 23.6 kg/m^2^, and 44.2% were current or ex-smokers. Of the study population, 34% were being treated for diabetes or satisfied the diagnostic criteria for diabetes, and 37.7% were hypertensive. Metabolic syndrome was present in 24.2%.

**Table 1:** Clinical characteristics of the study population.

N = 10,071	
Age (years)	48.7±12.8
Gender (male, %)	6475 (64.3)
Current or ex-smoker (%)	4451 (44.2)
Height (cm)	167.3±8.3
Weight (kg)	66.5±12.6
BMI (kg/m^2^)	23.6±3.3
Skeletal muscle mass (kg)	27.2±6
Body fat mass (kg)	17.5±5.9
Body fat (%)	26.2±6.6
Waist-hip ratio	0.9±0.1
Waist circumference (cm)	84.4±8.8
Hip circumference (cm)	93.9±7
Systolic blood pressure (mmHg)	121.6±13.3
Diastolic blood pressure (mmHg)	72.6±9.6
Hypertension (%)	3794 (37.7)
Diabetes (%)	3422 (34)
Metabolic syndrome (%)	2441 (24.2)
Laboratory Test	
Total cholesterol (mg/dL)	195±36.4
Triglyceride (mg/dL)	122.2±93.7
HDL-cholesterol (mg/dL)	51.7±13
LDL-cholesterol (mg/dL)	117.7±32.5
Fasting blood glucose (mg/dL)	98.8±21.5
HbA1_C_ (%)	5.6±0.7
Lung Function Test	
FEV_1_/FVC ratio	82.2±7.1
FVC % pred	91±11.2
FEV_1_% pred	97.7±13.8

Values are expressed as percentages, or mean ± standard deviation (SD). BMI = body mass index; HDL = high-density lipoprotein cholesterol; LDL = low-density lipoprotein cholesterol; HbA1c = hemoglobin A1c; FEV_1_ = forced expiratory volume in 1 s; FVC = forced vital capacity.

### Comparison of variables between the groups divided according to metabolic health and obesity status

A majority of (6,061, 60.2%) subjects were in MHNO group followed by 1,569 (15.6%), 804 (8.0%), 1,637 (16.3%) subjects classified into MHO, MUHNO, and MUHO group, respectively (Table 2).

**Table 2:** Comparison of variables between the groups divided according to metabolic health and obesity status.

	MHNO (%) n = 6,061 (60.2)	MHO (%) n = 1,569 (15.6)	MUHNO (%) n = 804 (8.0)	MUHO (%) n = 1,637(16.2)	P*
Age (years)	47.3±12.9^†^	47±12.2^†^	56.9±10.4	51.8±11.8	<0.001
Gender (male, %)	3299 (54.4)	1279 (81.5)	536 (66.7)	1361 (83.1)	<0.001
Current or ex-smoker (%)	2357 (38.9)	813 (51.8)	367 (45.6)	914 (55.8)	<0.001
Height (cm)	166.4±8.1^†^	168.9±8.1^‡^	166.2±8.8^†^	169.4±8.4^‡^	<0.001
Weight (kg)	60.3±9	76.6±9.2	64.5±8.2	80.3±11.5	<0.001
BMI (kg/m^2^)	21.7±2	26.8±1.8	23.3±1.4	27.9±2.6	<0.001
Systolic blood pressure (mmHg)	118.1±12.6	122.7±11.4	130.2±13.6^†^	129.5±12.2^†^	<0.001
Diastolic blood pressure (mmHg)	70.2±9.3	73.5±8.2	77.9±9^†^	78.2±8.8^†^	<0.001
Hypertension (%)	1428 (23.6)	499 (31.8)	636 (79.1)	1231 (75.2)	<0.001
Diabetes (%)	1240 (20.5)	341 (21.7)	666 (82.8)	1175 (71.8)	<0.001
Laboratory Test					
Total cholesterol (mg/dL)	192.9±34.7	199.9±34.9^†^	196.3±39.5^‡^	197.6±41.4^†^^‡^	<0.001
Triglyceride (mg/dL)	93.9±59.7	115±64.3	187.7±112.6	202.1±138.3	<0.001
HDL-cholesterol (mg/dL)	55.9±13.1	49.5±9.8	42.9±9.5^†^	42.9±9.7^†^	<0.001
LDL-cholesterol (mg/dL)	115.2±31.8^†^	126.8±31.2	117±33.8^†^^‡^	118.7±34^‡^	<0.001
Fasting blood glucose (mg/dL)	93.8±17.6	96.3±15.9	114.5±28.2	111.7±26.1	<0.001
HbA1c (%)	5.4±0.6	5.5±0.6	6±1.0	5.9±0.9	<0.001

Values are expressed as mean ± SD. MHNO = metabolically healthy non-obese; MHO = metabolically healthy obese; MUHNO = metabolically unhealthy non-obese; MUHO = metabolically unhealthy obese; BMI = body mass index; HDL = high-density lipoprotein cholesterol; LDL = low-density lipoprotein cholesterol; HbA1C = hemoglobin A1c.

*P values for one-way ANOVA among the four groups.

^†^,^‡^ No differences between the groups with same footnotes in post-hoc analysis.

The metabolically unhealthy groups (MUHNO and MUHO) were older compared to metabolically healthy peer groups. Mean BMI was approximately 22 kg/m^2^ in the non-obese groups and approximately 27 kg/m^2^ in the obese groups. Metabolically unhealthy groups had significantly higher proportions of subjects with diabetes and hypertension and showed higher fasting blood glucose, HbA1C, triglyceride and lower HDL-C levels compared to their metabolically healthy peers (*P* <0.001) All of the results were consistently significant even after adjustment for age and sex with ANCOVA test.

### Body composition variables in both sexes divided according to metabolic health and obesity status

Table 3 showed significant differences in body proportions among the four groups divided by sex (*P* <0.001). In females, MHO group had highest skeletal muscle mass (22.6±2.5 kg). The MUHO group had highest body fat mass (26.9±5.5 kg), body fat percentage (39.2±4.4%) and WHR (0.98). In males, MUHO group had the highest skeletal muscle mass (33.2±4.1 kg) and body fat mass (23.8±6.1 kg) followed by MHO group. WHR was also highest in the MUHO group (0.94).

**Table 3:** Body composition variables in both sexes divided according to metabolic health and obesity status.

**Female (n = 3,596)**	MHNO (%) n = 2,762 (76.8)	MHO (%) n = 290 (8.1)	MUHNO (%) n = 268 (7.4)	MUHO (%) n = 276 (7.7)	P*
Skeletal muscle mass (kg)	20.3±2.2^†^	22.6±2.5^‡^	20.1±2.2^†^	22.4±2.8^‡^	<0.001
Body fat mass (kg)	15.4±3.6	25.7±4.8	19.2±3.2	26.9±5.5	<0.001
Body fat (%)	28.7±5	38±4.1	33.7±4.2	39.2±4.4	<0.001
Waist-hip ratio	0.87±0.1	0.94^†^	0.94^†^	0.98	<0.001
Waist circumference (cm)	76.5±6.1	91.4±6.3	84.9±5.4	95±7.2	<0.001
Hip circumference (cm)	88.2±4.9	97.4±6.8^†^	89.9±4.6	97.6±6.6^†^	<0.001
**Male (n = 6,475)**	MHNO (%) n = 3,299 (50.9)	MHO (%) n = 1,279 (19.8)	MUHNO (%) n = 536 (8.3)	MUHO (%) n = 1,361 (21)	P*
Skeletal muscle mass (kg)	29.3±3.4^†^	32.7±3.7	29.1±3.3^†^	33.2±4.1	<0.001
Body fat mass (kg)	13.9±3.5	20.7±4.7	16.3±3.1	23.8±6.1	<0.001
Body fat (%)	20.9±4.4	26.3±4.4	23.8±3.8	28.6±4.6	<0.001
Waist-hip ratio	0.89	0.92	0.91	0.94	<0.001
Waist circumference (cm)	82.2±5	91.1±5.1	85.9±4.3	95.6±6.2	<0.001
Hip circumference (cm)	92.8±4.4	99.7±4.8	94.7±4.1	102.2±5.6	<0.001

Values are expressed as mean ± SD. MHNO = metabolically healthy non-obese; MHO = metabolically healthy obese; MUHNO = metabolically unhealthy non-obese; MUHO = metabolically unhealthy obese.

*P values for one-way ANOVA among the four groups.

^†^,^‡^ No differences between the groups with same footnotes in post-hoc analyses.

### Lung function among the groups divided according to metabolic health and obesity status

**Fig 1:**
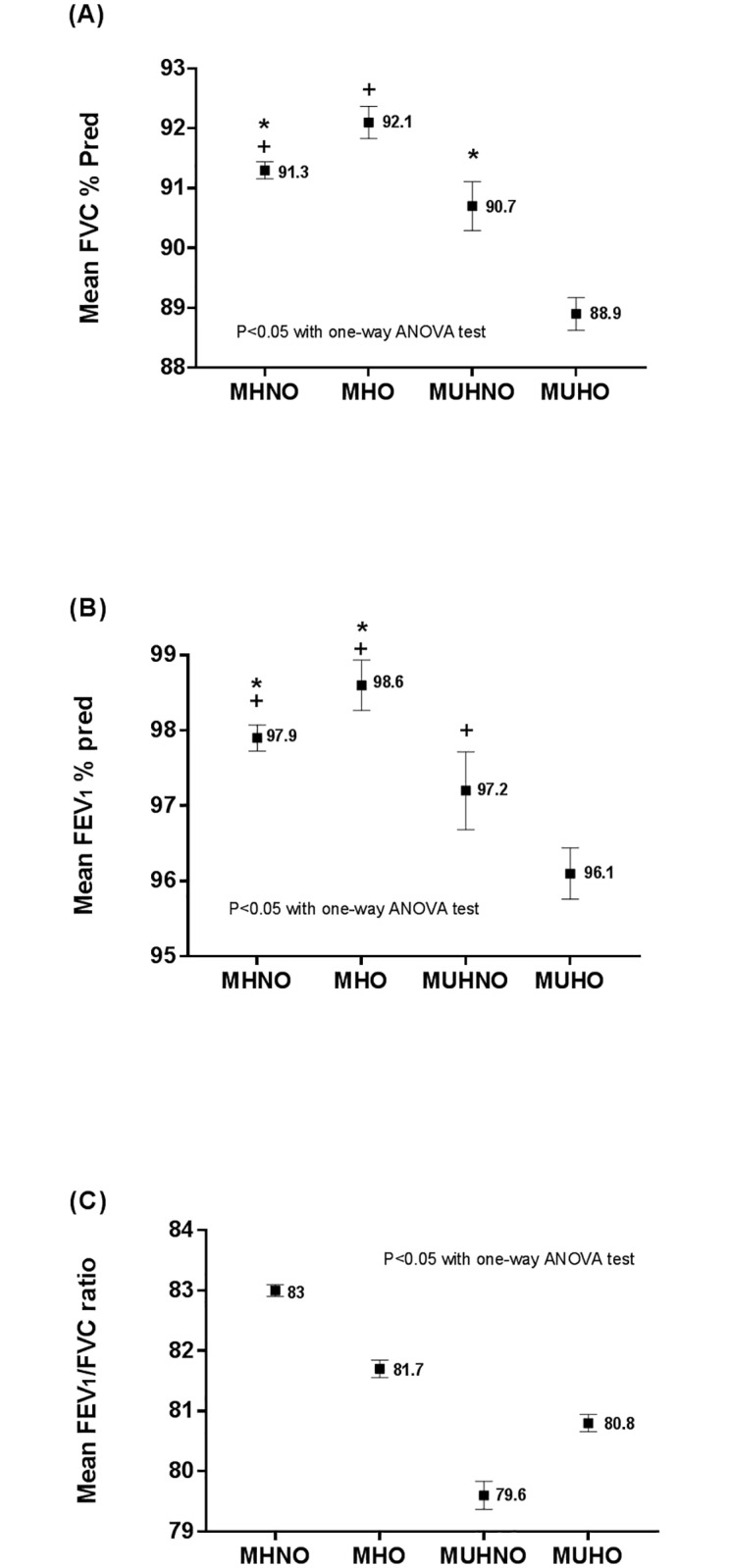
Lung function among the groups divided according to metabolic health and obesity status. (A) Mean FVC (% pred), (B) Mean FEV_1_ (% pred), (C) Mean FEV_1_/FVC ratio. FEV_1_ = forced expiratory volume in 1 s; FVC = forced vital capacity. *^, +^ Same footnotes denote no differences between the designated groups.

The MUHO group significantly had the lowest mean FVC (88.9±11.0, % pred) and FEV_1_ (96.1±13.8, % pred) values, meanwhile the MHO group significantly had the highest mean FVC (92.1±10.6, % pred) and FEV_1_ (98.6±13.3, % pred) values among the four groups. The MUHNO group had the lowest mean FEV_1_/FVC (79.6±6.6) values (Fig 1). Similar pattern was noted among the four groups when subgroup analysis in both non-smokers and ex-or current smokers was performed (S1 Table).

### Odds ratios for the decreased lung function and metabolic health status according to quartiles of FVC and FEV_1_% of predicted value

Multinomial logistic regression analysis with the quartiles of FVC or FEV_1_ (% pred) as the dependent variable and adjusted for age, sex, and smoking status was performed (model 1). Odds ratio (OR) for the lower FVC (<84% pred) was significantly higher in MUHO subjects (1.788 [95% CI, 1.531–2.089]) and lower in MHO subjects (0.768 [95% CI, 0.654–0.902]) with MHNO group as the reference, when OR for higher FVC (>98% pred) was considered as 1.0 (Table 4 Model 1). Odds ratio (OR) for the lower FEV_1_ (<89% pred) was significantly higher in MUHO subjects (1.603 [95% CI, 1.367–1.881]) and lower in MHO subjects (0.826 [95% CI, 0.700–0.976]) with MHNO group as the reference, when OR for higher FEV_1_ (>105% pred) was considered as 1.0 (Table 5 Model 1).

**Table 4:** Odds ratio for decreased lung function and metabolic health status according to quartiles of FVC % of predicted value.

	FVC (% pred) quartiles					
	1st (< 84)		2nd (84~90)		3rd (90–98)	
Variables	OR	95% CI	OR	95% CI	OR	95% CI
**Model 1**						
MHNO	1.000		1.000		1.000	
MHO	0.768	(0.654–0.902)	0.932	(0.795–1.092)	0.980	(0.846–1.134)
MUHNO	1.200	(0.976–1.477)	1.245	(1.000–1.550)	1.080	(0.879–1.328)
MUHO	1.788	(1.531–2.089)	1.487	(1.259–1.757)	1.270	(1.082–1.490)
**Model 2**						
MHNO	1.000		1.000		1.000	
MHO	0.702	(0.576–0.857)	0.820	(0.672–1.001)	0.915	(0.761–1.100)
MUHNO	1.100	(0.889–1.362)	1.157	(0.925–1.447)	1.040	(0.843–1.285)
MUHO	1.606	(1.294–1.995)	1.263	(1.005–1.587)	1.170	(0.943–1.453)

Model 1: Adjusted for age and sex and smoking. Model 2: Adjusted for Model 1 + skeletal muscle mass and body fat mass. FVC = forced vital capacity; OR = odds ratio; CI = confidence interval; MHNO = metabolically healthy non-obese; MHO = metabolically healthy obese; MUHNO = metabolically unhealthy non-obese; MUHO = metabolically unhealthy obese.

**Table 5:** Odds ratio for decreased lung function and metabolic health status according to quartiles of FEV_1_% of predicted value.

	FEV_1_ (% pred) quartiles					
	1st (< 89)		2nd (89~96)		3rd (97–105)	
Variables	OR	95% CI	OR	95% CI	OR	95% CI
**Model 1**						
MHNO	1.000		1.000		1.000	
MHO	0.826	(0.700–0.976)	0.937	(0.799–1.099)	1.050	(0.899–1.227)
MUHNO	1.421	(1.154–1.749)	1.248	(1.006–1.548)	1.193	(0.966–1.474)
MUHO	1.603	(1.367–1.881)	1.243	(1.053–1.468)	1.330	(1.131–1.563)
**Model 2**						
MHNO	1.000		1.000		1.000	
MHO	0.618	(0.505–0.757)	0.748	(0.615–0.910)	0.965	(0.797–1.168)
MUHNO	1.225	(0.990–1.516)	1.128	(0.905–1.405)	1.151	(0.928–1.427)
MUHO	1.093	(0.879–1.359)	0.922	(0.738–1.153)	1.191	(0.959–1.480)

Model 1: Adjusted for age and sex and smoking. Model 2: Adjusted for Model 1 + skeletal muscle mass and body fat mass. FEV_1_ = forced expiratory volume in 1 s; OR = odds ratio; CI = confidence interval; MHNO = metabolically healthy non-obese; MHO = metabolically healthy obese; MUHNO = metabolically unhealthy non-obese; MUHO = metabolically unhealthy obese.

When other parameters (model 1 + skeletal muscle mass and body fat mass) were adjusted in model 2, the OR were lowered but still showed similar trends with MUHO group showing the highest OR in the lower quartile of FVC (<84% pred), and MUHNO group showing the highest OR in the lower quartile of FEV_1_ (< 89% pred) (Tables 4 & 5).

## Discussion

From our study, metabolically unhealthy subjects were more prone to decreased lung function compared with their metabolically healthy counterparts, regardless of obesity status, suggesting that metabolic health is more associated with lung function impairment than obesity. OR for the lowest quartile of FVC and FEV_1_ (% pred) were significantly higher in metabolically unhealthy groups, even after adjusting for other metabolic parameters.

The term “metabolically healthy obesity (MHO)” comes from previous studies that observed a subgroup of obese subjects who do not have metabolic derangements or increased cardiometabolic risk. There is no unified consensus of metabolic health, as several different definitions have been used [5,6,8,23–25]. This has resulted in a wide range of prevalence (1.3–25.8%), clinical characteristics and outcomes [26]. In our study, the prevalence of MHO was similar to a large scale national Korean data (15.6 vs 15.2% of total subjects) [27]. Our data showed that MHO group had better lung function compared to metabolically unhealthy groups. This could be due to various reasons.

The MHO group had less individual components of MetS that are independently associated with lung function impairment. Percentage of underlying disease such as diabetes and hypertension that are known to be associated with decrease in lung function, was significantly lower in the MHO group compared to metabolically unhealthy groups. Elevated insulin levels can induce morphological or functional changes in airway smooth muscles and potentiate airway responsiveness which could reduce lung function [28–30]. Lee et al. showed that hypertension was also a risk factor for asthma like symptoms [31]. Presence of coronary artery calcium which implies atherosclerotic plaque burden, was an independent risk predictor for impaired lung function [32]. The MHO group had less components of dyslipidemia compared to metabolically unhealthy groups. Hyperlipidemia is known to activate fatty acid induced inflammation and is associated lung function impairment in adults and asthma risk in children [13,33,34]. Elevated triglyceride levels were also associated with airway hyperresponsiveness [35].

It is well known that risk factors such as hypertension, diabetes, high cholesterol levels, and obesity contribute to the development of cardiovascular disease [36]. Impaired lung function, such as reduced FEV1 and FVC is also another risk factor for cardiovascular morbidity and mortality [37]. FEV1 and FVC reduction even within normal range (from a mean of 109% to 88%, a value still, considered normal) is associated with cardiovascular disease risk [38]. Our study shows that metabolically unhealthy groups had significant lung function decline even in the normal range compared to the metabolically healthy groups.

Therefore our data is generally in line with previous reports that suggest an important role of MetS in decreased lung function. However regarding obesity, our result differed from previous studies [13]. It has been reported that abdominal adiposity can reduce expiratory reserve volume by compressing the lungs and diaphragm, leading to decrease in FVC [11,12]. Furthermore, systemic inflammation from visceral fat may also play a role in FVC decrease [39,40].

In our study, although the MHO and MUHO groups were both mostly in the moderate obesity (obese I, BMI 25~30) criteria, the MUHO group had the largest waist circumference, highest WHR and body fat mass resulting in the worst lung function among the four groups. This consistent with previous studies that show a significant negative correlation between body fat percentage and FVC or FVC/FEV_1_ [41]. Meanwhile the MHO group which had a larger waist circumference or body fat mass than MUHNO group, showed better lung function than both metabolically unhealthy groups. This suggests that the influence of metabolic health status is more associated with lung function than with obesity.

Despite having lower waist circumference and body fat mass, the MUHNO group showed similar WHR values and lower skeletal muscle mass compared to MHO group. Previous studies mention that not only the amount of fat mass but also the distribution of fat has been considered an important factor in determining lung function [42]. WHO states that WHR is a parameter to measure body fat distribution. The ratio can be measured more precisely than skin folds, and it provides an index of both subcutaneous and intra-abdominal adipose tissue [43,44]. Among WHR, BMI and body fat percentage, only the WHR takes account of the differences in body structure. In some studies, WHR has been found to be a more efficient predictor of cardiovascular disease and mortality than waist circumference and BMI [45,46]. Therefore, high WHR values which reflect increased amount of visceral adipose tissue, probably contributed to the decline in lung function in MUHNO group.

The reason for better lung function in the MHO group despite relative high WHR and body fat mass may be due to the difference in skeletal muscle mass. Skeletal muscle mass was highest in the MHO group among all groups in females. In males, the MHO group had second highest skeletal muscle mass. Skeletal muscle represents a large proportion of the fat-free mass of the body and is the most abundant insulin-sensitive tissue. Therefore sarcopenia (loss of skeletal muscle mass and/or muscle function) and age or obesity related skeletal muscle resistance to insulin may contribute to the metabolic dysregulation and the development of MetS [47]. Sarcopenia may be associated with pulmonary function not only in COPD and elderly patients with major comorbidities but also in healthy elderly men and women without lung disease diagnoses. Previous studies based on a large national Korean data showed that low muscle mass is an independent risk factor of decreased pulmonary function in healthy Korean men and women over 65 year of age [48,49].

Being metabolically unhealthy results in increased adipose tissue and reduced muscle mass which leads to lowering of lung function, whereas in the MHO group increase in skeletal muscle mass and less metabolic risk factors probably contributed to better lung function. Further investigations in the effect of different body structure and compositions on lung function are needed.

An interesting finding in our study was that the MHO group had better lung function than the MHNO group. Recently, the ‘obesity paradox’, which has been widely observed in obese subjects with different ethnicity, have demonstrated better survival compared to those with lower BMI levels [50–52]. Others argue that the ‘obesity paradox’ does not exist and have cited selection or survival bias, treatment bias, and other confounding variables as possible alternate explanations [53]. Our data suggests that the obesity paradox concept could be applied to lung function. This was a novel finding in our study. Favourable fat mass/fat-free mass ratio, nutritional status, cardiorespiratory fitness, greater likelihood of receiving optimal medical treatment, and cardioprotective metabolic effects of increased body fat have been suggested to explain the protective effect of obesity [52,54]. Further studies on other factors including cardiorespiratory fitness in this group remains to be studied.

To our knowledge, this is the first study that has analyzed the association between MHO and lung function in a large healthy population. Our study has strength that it is a large-scale, Asian study although conducted at a single center, and therefore selection bias would be low. However, there are limitations in our study. It was a retrospective study, thus time-dependent relationships between altered metabolic and obesity status with pulmonary function could not be observed. Also, we only used prebronchodilator data, our analyses may have included some percentage of participants with a reversible airways limitation.

In conclusion, in this large, health-screening population, metabolically unhealthy groups were more prone to decreased lung function compared with their metabolically healthy counterparts regardless of obesity status. Our findings suggest different approaches should be used in subjects with different metabolic health status, BMI and body composition, to improve lung function.

## Supporting information

S1 File Health questionnaire. Health questionnaire used in the health checkup programme at Seoul St. Mary’s Hospital.
(PDF)

S1 Table Lung function between ex or current smokers and non smokers divided according to metabolic health and obesity status. (DOCX)
