# Supplementary material for: Metabolic health is more closely associated with decrease in lung function than obesity
Source: PLoS One. 2019 Jan 23;14(1):e0209575. doi: 10.1371/journal.pone.0209575 (PMC6343891; doi:10.1371/journal.pone.0209575)
Supplement: S1 File — Health questionnaire used in the health checkup programme at Seoul St. Mary’s Hospital. (PDF) [file pone.0209575.s001.pdf]

Health Promotion Center
Health Renaissance for You

Health Questionnaire

**Date of Health check-up**	
**Hospital ID**	
**Name**	

※ Do you agree to receive health information or notices from the Seoul St.Mary's hospital?

(please check ☑) Yes □ No □

※ **Please complete the following questions about your present condition by ticking the appropriate box.**

1. **What is the main reason for a health screening?**
   ○ General Check-up ○ Employee's Check-up ○ Feel discomfort ○ Other

2. **Have you ever been diagnosed with a disease before?**
   ○ No ○ Yes (Name of diagnosis :)

3. **Have you had any kind of allergic reaction to certain food or medication?**
   ○ No ○ Yes (Name of food or medication :)

4. **Is there anything bothering you?**
   ○ No ○ Yes (Symptom :)

5. **Are you taking any kind of medication at present?**
   ○ No ○ Yes (Name of medication :)

6. **Have you had surgery before?**
   ○ No ○ Yes (Name of surgery :)

7. **Is there anybody among your family/relatives who has a serious illness or died from disease?**
   ○ No ○ Yes, Relationship ( ) Name of diagnosis ( )

8. **Do you Exercise? (to the point of perspiring)**
   ○ No ○ Yes (Name of exercise :)

9. **How often do you exercise?**
   ○ I don't exercise ○ 1~2 times ○ 3~4 times ○ Almost everyday

10. **How long do you exercise?**
    ○ Less than 15 minutes ○ 15~30 minutes ○ 30~60 minutes ○ More than 1 hour

11. **Do you drink alcohol? (What kind of alcohol/how much)**
    ○ No ○ Yes (_____time/week, Liquor/Beer/Wine ______bottles/glass/ 1time)

12. **Do you smoke?**
    ○ No ○ Smoke ○ Stop smoking ( ) years

13. **How much do you smoke a day?**
    ○ Less than half a pack ○ Half a pack ~ 1 pack ○ 1~2 pack ○ More than 2 packs

14. How long have you been smoking?

○ 1~5 years ○ 6~10 years ○ 11~20 years ○ more than 20 years

Classification	Questions		
Respiratory and Heart	I have cough and sputum(phlegm)	☐ Yes	☐ No
	I'm usually short of breath	☐ Yes	☐ No
	I have palpitations and irregular pulse	☐ Yes	☐ No
	I feel a squeezing pain in the chest	☐ Yes	☐ No
	I have difficulty breathing	☐ Yes	☐ No
	I feel pain when walking or exercising	☐ Yes	☐ No
Gastro-intestinal	I feel something stuck in my esophagus	☐ Yes	☐ No
	I have a hot, prickly feeling in the front of my chest	☐ Yes	☐ No
	I can't digest food well	☐ Yes	☐ No
	My stomach hurts when empty	☐ Yes	☐ No
	My stomach is bloated with gas when empty	☐ Yes	☐ No
	I have nausea and frequently vomit	☐ Yes	☐ No
	I have frequent bowel movements and my stool is very watery	☐ Yes	☐ No
	I alternate between diarrhea and constipation	☐ Yes	☐ No
	I have dark stool or have blood in the stool	☐ Yes	☐ No
	I have very thin stool	☐ Yes	☐ No
Genito-urinary	I get up frequently to urinate during the night	☐ Yes	☐ No
	I urinate often. (more than 6 times a day)	☐ Yes	☐ No
	I feel burning pain during urination	☐ Yes	☐ No
	I have difficulty urinating and have a sensation of not emptying after urination	☐ Yes	☐ No
	I can't hold my urine when my bladder is full	☐ Yes	☐ No
	I have had a sudden pain in the flank	☐ Yes	☐ No
	I have sexual dysfunction or disorders	☐ Yes	☐ No
Nervous system	I often have a headache	☐ Yes	☐ No
	I often feel very dizzy	☐ Yes	☐ No
	I am experiencing memory loss	☐ Yes	☐ No
	I have numbness in my hands and feet	☐ Yes	☐ No
	I sometimes have numbness or sensational abnormality in my arm or leg	☐ Yes	☐ No
	I have lost my consciousness before	☐ Yes	☐ No
**Classification**	**Questions**		
Other	Weight gain (more than 6kg in 6months)	☐ Yes	☐ No

	Weight loss (more than 6kg in 6months)	☐ Yes	☐ No
	I feel tired and get fatigued easily	☐ Yes	☐ No
	I can't tolerate heat or thirst	☐ Yes	☐ No
	I bruise easily	☐ Yes	☐ No
	I can't concentrate, and I feel irritated	☐ Yes	☐ No
	I am always nervous and restless	☐ Yes	☐ No
	I don't like meeting people	☐ Yes	☐ No
	I often feel depressed	☐ Yes	☐ No
	I feel a lump in the neck, armpits, groin area	☐ Yes	☐ No
	I often wake up at night and can't sleep soundly	☐ Yes	☐ No
Women's problems	Marital status (single married)	☐ Yes	☐ No
	Pulmonary disease	☐ Yes	☐ No
	Irregular period	☐ Yes	☐ No
	I have abnormal bleeding	☐ Yes	☐ No
	I have too much vaginal discharge	☐ Yes	☐ No
	I have a lump in my breast	☐ Yes	☐ No
	Lately I have discharge such as blood or water in my nipples	☐ Yes	☐ No
	Is there history of breast cancer among your family or relatives?<br>①None ②Either aunt or grandmother ③Either mother or sister		
Do you agree to the test for HIV?	☐ Yes Signature( ), ☐ No		

Thank you for answering the health questionnaire.
