# Supplementary material for: Metabolic health is more closely associated with decrease in lung function than obesity
Source: PLoS One. 2019 Jan 23;14(1):e0209575. doi: 10.1371/journal.pone.0209575 (PMC6343891; doi:10.1371/journal.pone.0209575)
Supplement: S1 Table — (DOCX) [file pone.0209575.s002.docx]

| **Ex or current smoker** |  |  |  | |  |  |  |  |  |
| --- | --- | --- | --- | --- | --- | --- | --- | --- | --- |
|  | Total  (n=4,451, 44.2%) | MHNO (%) n=2,357 (53) | MHO (%)  n=813 (18.3) | | MUHNO (%) n=367 (8.2) | MUHO (%)  n=914 (20.5) | *P** |  |  |
| Lung Function Test |  |  |  | |  |  |  |  |  |
| FEV1/FVC ratio (%) | 81±7.1 | 81.5±7.9† | 81.1±5.7† | | 79.3±7.1 | 80.4±5.8 | <0.001 |  |  |
| FVC % pred | 91±10.8 | 91.7±10.7^†^ | 91.8±10.6^†^ | | 90.1±11.3 | 88.6±10.8 | <0.001 |  |  |
| FEV_1_% pred | 96.7±13.2 | 97.2±13.3^†^ | 97.3±12.7^†^ | | 96±13.8^†‡^ | 94.8±13.2^‡^ | <0.001 |  |  |
|  |  |  |  | |  |  |  |  |  |
|  |  |  |  | |  |  |  |  |  |
| **Non smoker** |  |  |  | |  |  |  |  |  |
|  | Total  (n=5,620, 55.8%) | MHNO (%) n=3,704 (65.9) | | MHO (%)  n=756 (13.4) | MUHNO (%) n=437 (7.8) | MUHO (%)  n=723 (12.9) | *P** |  |  |
| Lung Function Test |  |  |  | |  |  |  |  |  |
| FEV1/FVC ratio (%) | 83.1±7 | 83.9±7.3 | 82.4±5.7 | | 79.9±6.1 | 81.3±5.9 | <0.001 |  |  |
| FVC % pred | 91±11.5 | 91.1±11.5^†^ | 92.5±10.7 | | 91.0±11.9^†^ | 89.3±11.3 | <0.001 |  |  |
| FEV_1_% pred | 98.5±14.1 | 98.3±14^†^ | 99.9±13.8 | | 98.3±15.4^†^ | 97.8±14.3^†^ | 0.018 |  |  |

Values are expressed as mean ± SD. MHNO = metabolically healthy non-obese; MHO = metabolically healthy obese; MUHNO = metabolically unhealthy non-obese; MUHO = metabolically unhealthy obese; FEV_1_ = forced expiratory volume in 1 s; FVC = forced vital capacity.

**P* values for one-way ANOVA among the four groups.

†‡ No differences between the groups with same footnotes in post-hoc analyses.
